# Integrated multi-tissue transcriptomic and network analyses reveal systemic crosstalk governing the onset of lay in Wuhua Yellow chickens

**DOI:** 10.1016/j.psj.2026.108073

**Published:** 2026-10-31

**Authors:** Yong-Jie Xu, Yi-Xuan Wang, Zhuo-Xian Weng, Yu-Nan Zou, Wei-Na Li, Jie-Bo Chen, Bing-Wang Du, Xun-He Huang

**Affiliations:** Guangdong Provincial Key Laboratory of Conservation and Precision Utilization of Characteristic Agricultural Resources in Mountainous Areas, Guangdong Innovation Centre for Science and Technology of Wuhua Yellow Chicken, School of Life Sciences, Jiaying University, Meizhou, 514015, China

**Keywords:** Onset of lay, HPO–liver axis, Multi-tissue transcriptomics, WGCNA, Ligand–receptor interaction

## Abstract

The onset of lay in poultry is a systemic transition requiring tight neuroendocrine–metabolic coordination. Although indigenous breeds retain valuable genetic diversity and resilience, their multi-tissue regulatory architecture remains poorly understood. Here, we integrated transcriptomes across four key tissues (hypothalamus, pituitary, ovary, and liver) in Wuhua Yellow chickens at pre-laying (16 weeks) and peak-laying (32 weeks) stages, combining weighted gene co-expression network analysis, ligand–receptor inference, and quantitative real-time PCR (**qPCR**) validation. Results support a coordinated HPO–liver expression program rather than isolated organ responses. Systemic prolactin (***PRL***) induction was coupled with hepatic *PRLR* upregulation (log_2_FC = 4.23), coinciding with massive hepatic induction of yolk precursors: *VTG1* (log_2_FC = 18.28), *VTG2* (log_2_FC = 6.60), and *APOB* (log_2_FC = 3.31). Reciprocal downregulation of pituitary lipoprotein receptors (*VLDLR* and *LRP2*) suggested a possible feedback-related route consistent with peripheral metabolic status. WGCNA identified tissue-specific hub genes associated with a functional division of labor across the axis, including hypothalamic (*ADGRL3, NRXN1*), pituitary (*EYA1*), ovarian (*MAD2L1, PLK1*), and hepatic (*HGFAC, PAH*) modules. Significant negative inter-module correlations between liver and central/gonadal tissues (*r* = −0.48 to −0.68, *P* < 0.05) highlighted a systemic trade-off reallocating resources toward yolk synthesis. Targeted qPCR confirmed strong stage-dependent changes in core signaling and yolk-precursor genes (*PRL, VTG1, VTG2, APOB*, and *SPP1*) and a significant peak-lay decrease in hepatic *PAH*, whereas other representative hubs (*NRXN1, EYA1*, and *PLK1*) tracked RNA-seq trends. Overall, this multi-tissue framework provides a transcriptomic-based framework for understanding stage-dependent transcriptional coordination of reproductive activation in an indigenous chicken and nominates expression-level candidate genes for further genetic and functional validation toward improving egg production while preserving native genetic diversity.

## Introduction

Egg production is a core economic trait in poultry and a complex physiological outcome of coordinated neuroendocrine, ovarian and metabolic regulation (Tan et al., 2025). In commercial layers, decades of intensive selection have driven high laying rates (typically >300 eggs per year), whereas most indigenous Chinese breeds exhibit comparatively modest egg yields (often <150 eggs per year), despite retaining valuable genetic diversity, stress resilience and superior meat quality (Weng et al., 2020; Huang et al., 2025; National Commission for Animal Genetic Resources, 2025). Understanding how the onset of lay is systemically organized at a multi-tissue level in such unselected populations is therefore essential both for unraveling core reproductive biology and for guiding the sustainable genetic improvement of dual-purpose local chickens.

The classical hypothalamic–pituitary–ovarian (**HPO**) axis initiates reproductive activation through gonadotropin-releasing hormone (**GnRH**), gonadotropins and ovarian steroid feedback (Xu et al., 2023; Li et al., 2025). In birds, however, successful egg formation also depends on the liver, which synthesizes and secretes the major yolk precursors vitellogenin (***VTG***) and apolipoprotein B (***APOB***)–containing lipoproteins under estrogenic and related endocrine control (Gloux et al., 2019; Ren et al., 2021). Prolactin (***PRL***) and its receptor (***PRLR***) further modulate reproductive state and hepatic metabolic responsiveness during laying transitions (Gloux et al., 2019; Chen et al., 2024). Thus, the functional unit governing the transition from the pre-laying to the peak-laying stage can be conceptualized as an extended HPO–liver axis rather than an isolated HPO axis alone (Shi et al., 2024; Wang et al., 2024).

Recent transcriptomic and multi-omics studies have begun to map components of this axis. Hypothalamic and pituitary RNA-seq profiling has linked neuropeptides, *PRL* and gonadotropin-related genes to laying stage or yield differences (Ellestad et al., 2019; Xu et al., 2023; Chen et al., 2024; Li et al., 2024). Ovarian profiles highlight follicle development, cell-cycle control and tissue remodeling associated with maturation and production level (Jia et al., 2023; Leng et al., 2024). Hepatic studies have identified *VTG, APOB* and lipid-metabolic pathways as hallmarks of peak anabolic demand (Gloux et al., 2019; Ren et al., 2021). Furthermore, whole-transcriptome analyses across the HPO axis, including non-coding RNAs, have illustrated multi-layered regulation of reproduction (Li et al., 2025). Genome-wide association studies (**GWAS**) and multi-tissue communication analyses have likewise nominated candidates for egg number, age at first egg and related traits, often implicating hormone signaling, cell adhesion and metabolic loci (Wang et al., 2024; Tan et al., 2025; Zhu et al., 2025).

Despite these advances, important gaps remain. Many studies focus on a single tissue, compare high- versus low-yield lines at one adult age, or focus primarily on commercial rather than indigenous genetics; consequently, stage-dependent crosstalk at the true onset of lay remains incompletely resolved (Wang et al., 2024; Zhu et al., 2025). Additionally, ligand–receptor communication between central and peripheral organs is often inferred from circulating hormone assays or candidate genes rather than decoded from genome-wide, multi-tissue expression networks. While weighted gene co-expression network analysis (**WGCNA**) can identify tissue-specific hubs and inter-module relationships (Langfelder and Horvath, 2008), the integration of co-expression topology with intercellular signaling maps and orthogonal quantitative real-time PCR (**qPCR**) validation remains exceptionally limited in indigenous poultry.

The Wuhua Yellow chicken (**WYC**), an indigenous dual-purpose breed from Guangdong, China, exemplifies this challenge and opportunity. Genomic surveys have described its population structure, genetic variation and selection signatures (Weng et al., 2020). A recent GWAS in the same population identified candidates for egg-laying performance, including loci related to follicular development and metabolism (Huang et al., 2025). Moreover, ovarian m^6^A and transcriptome profiling have linked epitranscriptomic regulation to sexual maturation and moderate low egg output (Jia et al., 2023). These resources establish a solid genetic and molecular baseline for WYCs; however, a simultaneous, four-tissue perspective (hypothalamus, pituitary, ovary, and liver) of the pre-laying to peak-laying transition, and of the inter-tissue communication routes that couple central neuroendocrine signals to peripheral anabolic output, remains lacking.

Here, we performed RNA-sequencing (**RNA-seq**) on four key tissues (hypothalamus, pituitary, ovary and liver) of WYCs at pre-laying (16 weeks) and peak-laying (32 weeks) stages, integrating differential expression analysis, global WGCNA, multi-stage ligand–receptor communication inference, and targeted qPCR validation. Unlike studies that compare high- versus low-yielding adult hens at a single reproductive stage, we focused on the pre-lay to peak-lay transition within an unselected indigenous population. Specifically, we aimed to: (i) define systemic transcriptional remodeling at the onset of lay, (ii) identify tissue-specific co-expression hubs that encode the functional division of labor across the HPO-liver axis, and (iii) map inter-tissue ligand–receptor axes linking central endocrine output to hepatic yolk precursor synthesis and ovarian reception using expression-based inference from orthology-mapped interaction databases. The resulting HPO–liver framework provides mechanistic insight into reproductive activation in an indigenous chicken and nominates expression-level candidate genes for further genetic and functional validation toward improving egg production while preserving genetic diversity.

## Materials and Methods

### Ethical approval and animal husbandry

All procedures were approved by the Animal Ethics and Welfare Committee of Jiaying University (Approval No.: JYYXLL2025-13). Healthy female WYCs were reared under identical management at Fengdu Poultry Farm (Meizhou, Guangdong, China) with ad libitum access to feed and water (16L:8D photoperiod). Based on continuous oviposition monitoring, birds were selected at two critical stages (*n* = 6 per group): (1) Pre-laying (**Pre**): 16-week-old hens prior to lay with uniform body weight; (2) Peak-laying (**Peak**): 32-week-old top 10% producers (laying rate > 90%). Three representative hens per group were designated for RNA-seq (*n* = 3 per tissue–stage, 24 total libraries) (Table S1), while all six birds per group (*n* = 6) were used for qPCR validation. An overview of the experimental design and analytical workflow is provided in Fig. 1.

**Fig. 1:**
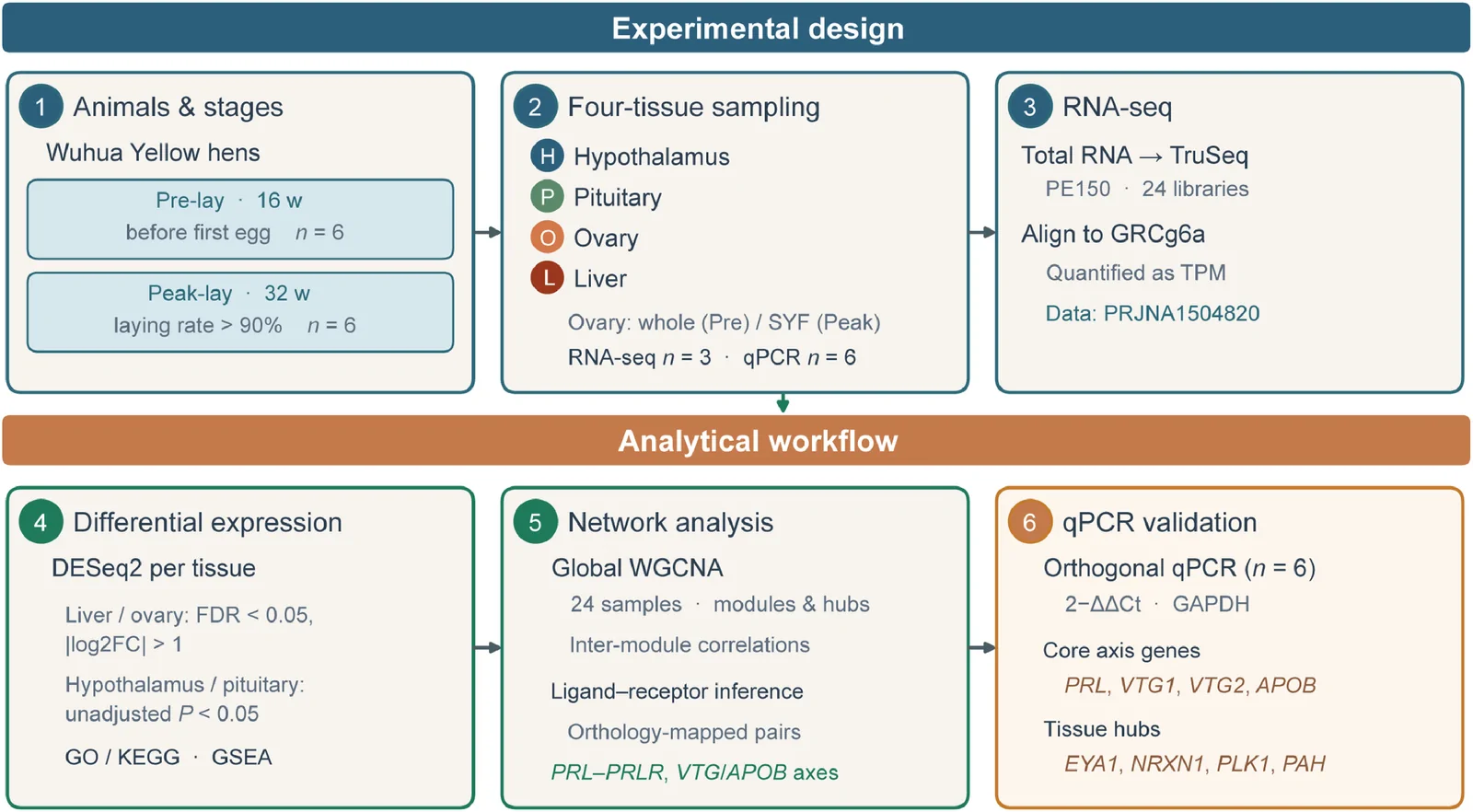
**Experimental design and analytical workflow of the multi-tissue transcriptomic study in Wuhua Yellow chickens**. Hens at the pre-laying (16 weeks) and peak-laying (32 weeks) stages (*n* = 6 per stage) were sampled for hypothalamus, pituitary, ovary, and liver. RNA-seq (*n* = 3 per tissue–stage) was followed by tissue-stratified differential expression analysis, GO/KEGG enrichment, GSEA, global WGCNA, and ligand–receptor inference. Key findings were orthogonally validated by qPCR (*n* = 6).

### Tissue Harvesting and Preparation

Birds were humanely stunned and exsanguinated via jugular venipuncture to harvest hypothalamus (H), pituitary (P), ovary (O), and liver (L) tissues. Specifically, whole ovaries containing all immature follicles were collected at the pre-laying stage due to the absence of morphologically distinguishable follicle hierarchies, whereas small yellow follicles were isolated at peak-lay. Harvested tissues were rinsed with ice-cold PBS, snap-frozen in liquid nitrogen, and stored at −80°C until RNA extraction.

### Transcriptomic sequencing and data analysis

To profile the transcriptomic dynamics across different laying stages, total RNA was extracted from the collected tissue samples using TRIzol® Reagent (Invitrogen, USA) according to the manufacturer's instructions. RNA integrity and concentration were assessed using an Agilent 2100 Bioanalyzer (Agilent Technologies, Santa Clara, CA, USA) and a NanoDrop 2000 spectrophotometer (Thermo Fisher Scientific, Waltham, MA, USA). A total amount of 1 μg RNA per sample was used as input material for RNA sample preparations.

Sequencing libraries were generated using an Illumina TruSeq RNA Library Prep Kit following the manufacturer's recommendations. Paired-end sequencing (150-bp reads) was subsequently performed on an Illumina NovaSeq X Plus platform at Gene Denovo Biotechnology Co., Ltd. (Guangzhou, China). Raw reads containing adapters or low-quality bases were filtered using FASTP v0.20.0 (Chen, 2023). Clean reads were then aligned to the chicken reference genome GRCg6a (assembly accession: GCA_000002315.5) using HISAT2 v2.1.0 (Pertea et al., 2016) and assembled using STRINGTIE v1.3.1 (Pertea et al., 2016). Gene expression levels were quantified and normalized as transcripts per kilobase million (**TPM**) using RSEM v1.3.1 (Li and Dewey, 2011). Differential expression analysis between the Peak and Pre groups was performed independently for each of the four tissues (hypothalamus, pituitary, ovary, and liver) using the DESeq2 v1.36.0 (Love et al., 2014). A stratified thresholding strategy was used to account for tissue-specific heterogeneity. For peripheral tissues (liver and ovary), differentially expressed genes (**DEGs**) were identified using stringent criteria (FDR < 0.05 and |log2FC| > 1). For the highly heterogeneous neuroendocrine tissues (hypothalamus and pituitary), DEGs were defined using an unadjusted *P* < 0.05 to retain subtle yet critical regulatory hubs, with downstream robustness ensured through WGCNA and orthogonal qPCR validation. Functional enrichment analyses, including Gene Ontology (**GO**) and Kyoto Encyclopedia of Genes and Genomes (**KEGG**) pathway analyses, were subsequently conducted using KOBAS v3.0 (Bu et al., 2021).

Gene Set Enrichment Analysis (**GSEA**) was performed using GSEApy (v1.0.5) (Fang et al., 2023) against MSigDB v2023.1 (C2 and C5 gene sets) to identify pathway-level changes between phenotypic groups. A |Normalized Enrichment Score (**NES**)| > 1, a nominal *P*-value < 0.05, and an FDR Q-value < 0.25 were used as criteria for significant enrichment.

### WGCNA

WGCNA was conducted using the WGCNA R package (Langfelder and Horvath, 2008) on qualified genes (TPM ≥ 1) across 24 samples (4 tissues × 2 stages × 3 replicates). A soft-thresholding power of *β* = 8 was selected to establish a scale-free network topology (*R*^2^ > 0.85). Modules were defined via dynamic tree cutting with a minimum size of 30 genes and a merge cut height of 0.25. Pearson correlations were computed between module eigengenes (**MEs**) and traits/tissues. Candidate hub genes were screened using Module Membership (**MM** > 0.9) and Gene Significance (**GS** > 0.9). Top topological overlap matrix (TOM)-weighted interactions (weight > 0.15) were exported to Cytoscape (v3.10.4) (Shannon et al., 2003) for network visualization.

### Protein-protein Interaction (PPI) network construction and module Analysis

PPI networks were constructed via the STRING database (v11.5) (Szklarczyk et al., 2023), restricted to *Gallus gallus* with a medium confidence score threshold (> 0.400). The integrated network was visualized and topologically analyzed in Cytoscape (v3.10.4). Key hub nodes were identified using the CytoHubba plugin based on the Degree algorithm (degree ≥ 30). Densely connected functional sub-clusters were extracted using the MCODE plugin (score ≥ 4.0; degree cutoff = 2, node score cutoff = 0.2, k-core = 2, max depth = 100). Downstream KEGG pathway enrichment was performed on module-assigned genes to pinpoint tissue-specific functional drivers.

### Inter-tissue ligand–receptor communication analysis

Potential ligand–receptor (**L–R**) pairs were retrieved from CellChatDB (Jin et al., 2021) and mapped to *Gallus gallus* orthologs via Ensembl BioMart (high-confidence orthologs only). Biologically prioritized reproductive–metabolic axes (e.g., PRL–PRLR, VTG/APOB–lipoprotein receptors, NPY-, VIP-, GAL-, and SPP1–CD36-related pairs) were retained. For each pair, mean TPM was calculated per tissue–stage, and stage regulation was summarized as log_2_FC (peak vs pre) with FDR from Section 2.3. Communication routes were defined as source tissue → ligand → receptor → target tissue. Interaction strength was quantified from peak-laying ligand and receptor expression levels and used to scale connection weights in the network. Sankey diagrams were drawn with plotly, with flow widths proportional to these peak-laying weights and colors by tissue origin.

### qPCR Validation

To validate the reliability of the RNA-seq data and confirm the expression shifts of key functional nodes, qPCR was performed on key candidate genes across representative tissues (*n* = 6 biological samples per tissue group). First-strand cDNA was synthesized from 1 μg of total RNA using a PrimeScript^TM^ RT Reagent Kit with gDNA Eraser (Takara, Dalian, China) according to the manufacturer's instructions. Specific primers for the selected candidate genes and the reference gene *GAPDH* were designed online using the NCBI Primer-BLAST tool (https://www.ncbi.nlm.nih.gov/tools/primer-blast/) (Table S2). Real-time PCR reactions were performed on a MA-6000 system (Suzhou Yairui Biological Technology Co., Ltd., Suzhou, China) using SYBR Green Premix Ex Taq^TM^ II (Takara, Dalian, China). Each reaction was conducted in a 20 μL volume containing 2 μL of diluted cDNA, 10 μL of SYBR Green Mix, 0.8 μL of each forward and reverse primer, and 6.4 μL of nuclease-free water. The thermal cycling conditions consisted of an initial denaturation at 95°C for 30 s, followed by 40 cycles of 95°C for 5 s and 60°C for 30 s. Relative expression levels of the target genes were calculated using the 2^−ΔΔCt^ method, with normalization against the reference gene. All reactions were performed in triplicate for each biological sample. Statistical significance between Pre and Peak groups was assessed by two-tailed Student’s *t*-test, with *P* < 0.05 considered significant.

## Results

### Phenotypic and systemic transcriptomic overview of the onset of lay

Ovaries from pre-laying WYCs exhibited undeveloped morphology with small, undifferentiated follicles, whereas those from peak-laying birds displayed fully developed hierarchical follicles (Fig. 2A, B), illustrating a dramatic physiological transition associated with reproductive maturation.

**Fig. 2:**
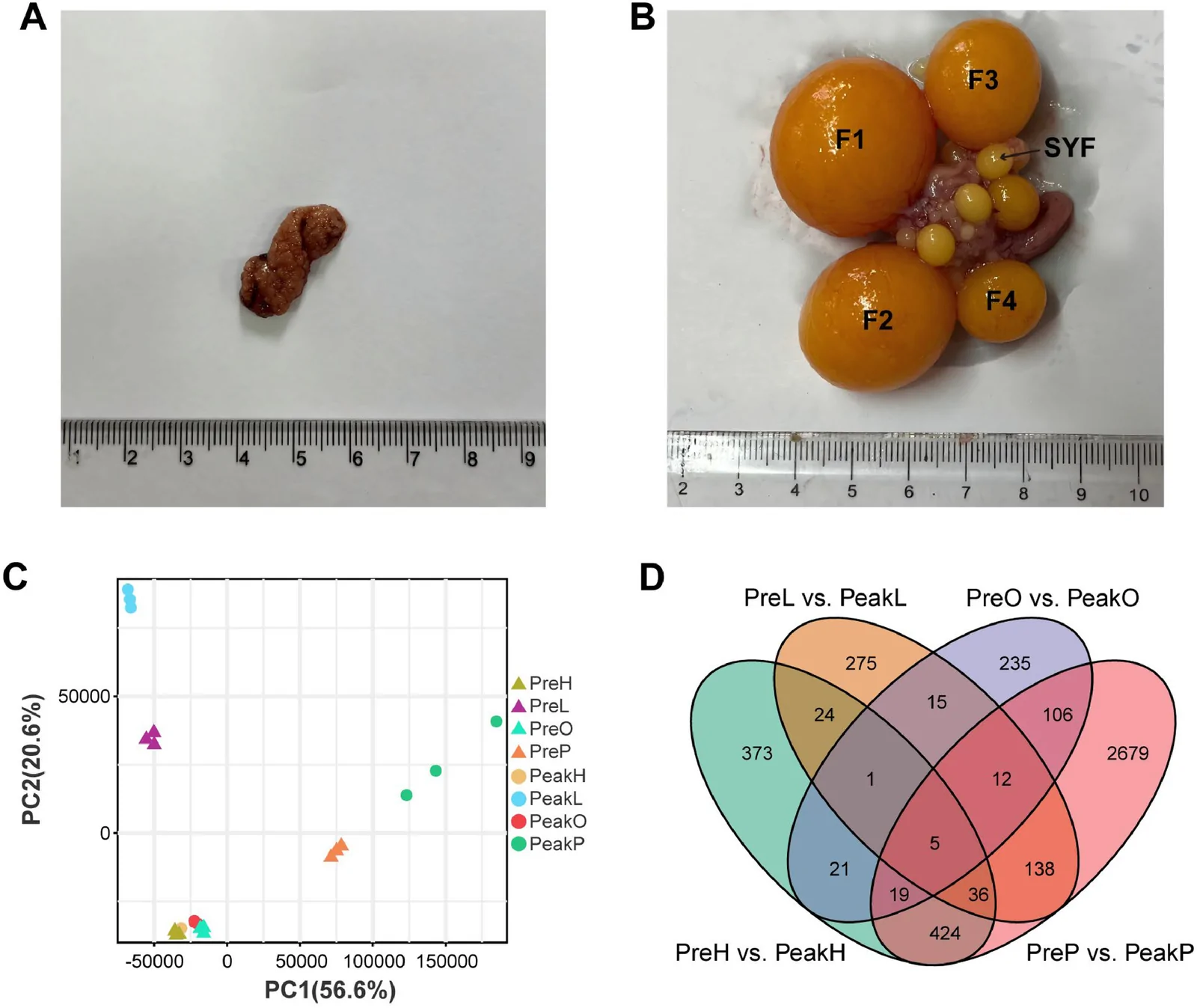
**Phenotypic and global transcriptomic overview of WYCs at pre-laying and peak-laying stages**. (A, B) Ovarian morphology of WYCs at the pre-laying (A) and peak-laying (B) stages (scale bar = 1 cm). In panel B, hierarchical follicles (F1–F4) and small yellow follicles (SYF) are labeled. (C) Principal component analysis (PCA) of global gene expression profiles across the hypothalamus, pituitary, ovary, and liver tissues between the pre-laying (triangles) and peak-laying (circles) groups. (D) Venn diagram illustrating unique and shared differentially expressed genes across tissues (PreH vs. PeakH, PreP vs. PeakP, PreO vs. PeakO, and PreL vs. PeakL). Abbreviations: H, hypothalamus; P, pituitary; O, ovary; L, liver; SYF, small yellow follicles.

To elucidate the molecular basis of this transition at a systemic level, we performed transcriptome sequencing on the hypothalamus, pituitary, ovary, and liver tissues. Quality control analysis confirmed the generation of high-quality sequencing data across all samples, with raw data yields ranging from 5.4 to 7.9 Gb, Q30 percentages exceeding 94.85%, and total mapping rates above 92.85% (Table S1). Furthermore, principal component analysis (**PCA**, Fig. 2C) demonstrated a clear separation between the pre-laying and peak-laying groups across all four tissues, indicating profound stage-specific transcriptional reprogramming throughout the neuroendocrine-reproductive-metabolic axis.

### Central transformation and functional specialization in the reproductive axis center

Using a stratified thresholding strategy, we identified a total of 903 DEGs in the hypothalamus, 3419 in the pituitary, 414 in the ovary, and 506 in the liver (Tables S3−S6). Venn diagram analysis revealed both tissue-specific and shared transcriptional responses among these organs during the transition from the pre-laying to the peak-laying stage (Fig. 2D). Notably, *VTG2* was among a small set of genes detected as differentially expressed in all four tissues and showed the strongest upregulation in the liver (log_2_FC = 6.60).

The hypothalamus and pituitary exhibited close transcriptional coupling, sharing 484 common DEGs that reflect coordinated neuroendocrine remodeling (Fig. 2D). Key central network hubs (*NAV3, KIDINS220*, and *NPY*) showed synchronized expression shifts. Furthermore, a subset of 24 genes was concurrently regulated across the hypothalamus, pituitary, and ovary, establishing a tri-tissue axis. Notably, *PRL* showed a marked hypothalamic increase (log_2_FC = 7.77), and local *GRP* upregulation further underscored the integration of energy balance and reproductive activation.

The pituitary displayed the most extensive transcriptomic changes (3,419 DEGs), reflecting a major functional specialization (Table S4). Core reproductive pathways were activated via genes such as *EGR1*, while *ESR1* was suppressed, consistent with adjusted feedback control. Transcripts of the yolk-precursor genes *VTG1* and *VTG2*, which are classically associated with hepatic expression, were also detected as up-regulated in the pituitary at peak-lay; however, their absolute expression levels remained far lower than in the liver, and the biological relevance of this low-level detection warrants caution pending protein-level and cell-type-resolved validation. Concurrently, substantial downregulation of structural regulators, such as *MYL2* (log_2_FC = −6.13), indicated that the pituitary transitions into a functionally mature operational state optimized for peak lay.

### Functional synergy and division of labor in terminal organs: ovary and liver

As the two principal execution terminals of the reproductive process, the ovary and liver exhibited functional synergy and division of labor during peak lay. In the ovary, DEGs orchestrated follicular development and ovulation (Table S5). This included a burst of yolk deposition, characterized by the significant upregulation of VTG2 (log_2_FC = 5.93) and APOB (log₂FC = 4.89). Steroid hormone synthesis underwent reconfiguration, evidenced by the downregulation of CYP17A1 and STAR, revealing adjusted hormonal regulation for resource allocation. Furthermore, the robust upregulation of matrix metalloproteinase 13 (MMP13, log_2_FC = 5.20) and various collagens indicated extensive extracellular matrix (**ECM**) remodeling, which is essential for structural growth and follicular rupture during ovulation.

Concurrently, the hepatic transcriptomic profile revealed a specialized metabolic state (Table S6). The synthesis of yolk precursor was markedly activated, with VTG1 exhibiting a substantial upregulation (log_2_FC = 18.28), which, alongside VTG2 and APOB, constituted the primary components for egg production. Lipid metabolism was significantly reconFig.d to support this demand: the liver maintained a continuous substrate flow by upregulating lipid mobilization genes (e.g., ABHD5), downregulating lipid retention genes (e.g., LPL, log_2_FC = −6.35), and modulating cholesterol trafficking. Enhanced hormonal sensitivity, indicated by the upregulation of PRLR and PAQR7, together with the suppression of catabolic genes such as IDO2, is consistent with a metabolic redirection toward reproductive dominance.

### Functional pathways and GSEA basis of multi-tissue synergy

#### Central neuroendocrine regulation: hypothalamus-pituitary

To elucidate the central neuroendocrine mechanisms governing the transition from pre-laying to peak-laying stages, functional enrichment analyses of DEGs were integrated across the hypothalamus-pituitary axis. In the hypothalamus, functional annotation and GSEA together revealed a highly integrated sensing and signaling hub that synchronizes environmental and metabolic inputs to trigger neuroendocrine cascades. Rather than acting merely as a passive hormone emitter, the hypothalamus aligned circadian photoperiodic gating (*CLOCK, NPAS2*) and energy homeostasis (*NPY, ADIPOR2*) to drive downstream neuroendocrine outputs (Table S7). This central signaling was anchored by GPCR-mediated receptor networks, led by the Neuroactive ligand-receptor interaction hub (featuring core neuropeptides and receptors *PRL, NPY, VIP, CCRH*; NES = 1.95, FDR < 0.01), which further engaged downstream Calcium, MAPK, and GnRH cascades to initiate the central reproductive axis (Fig. 3A, B; Table S8).

**Fig. 3:**
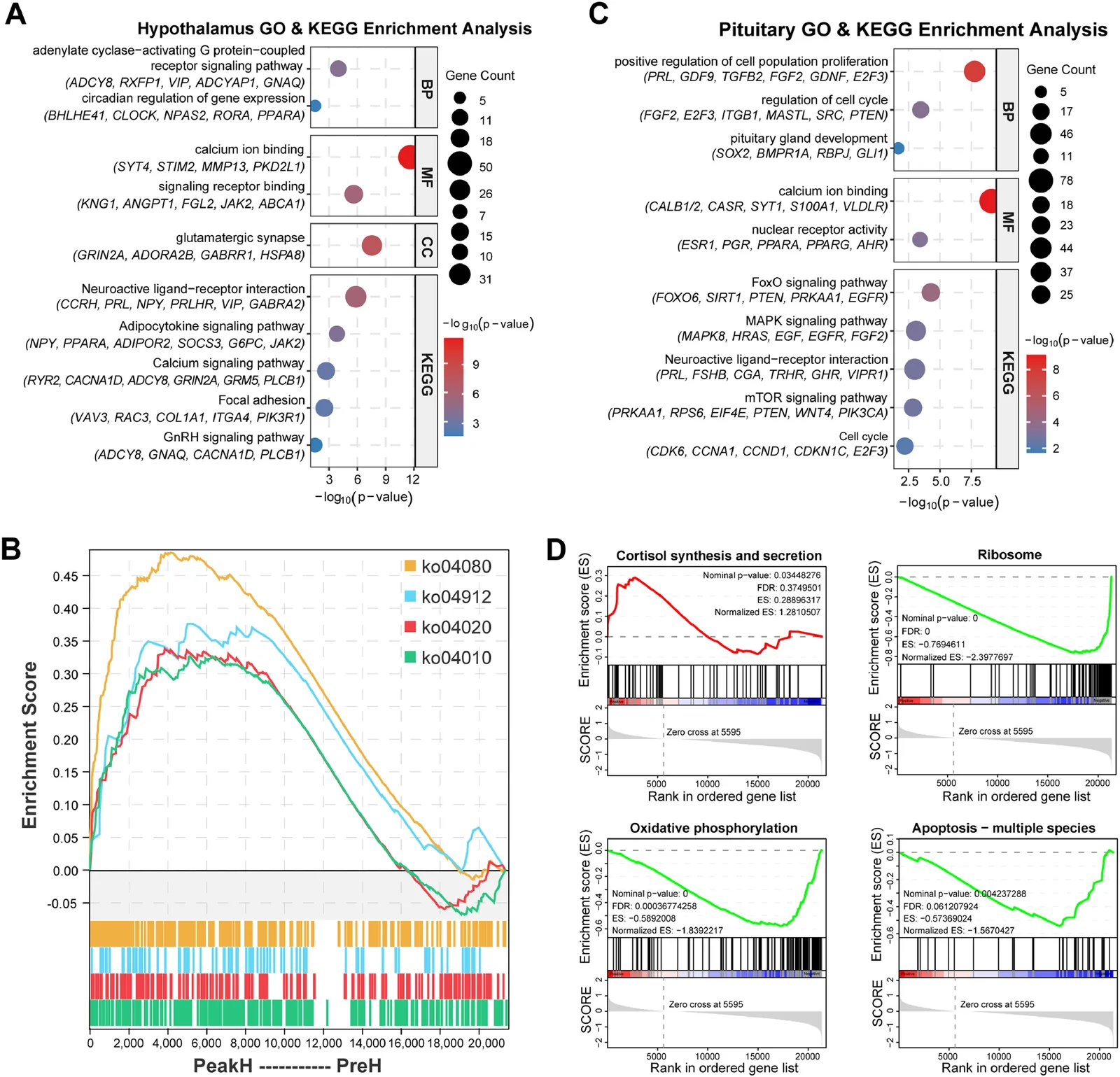
**Functional enrichment analysis of differentially expressed genes (DEGs) and gene set enrichment analysis (GSEA) in the hypothalamus and pituitary**. (A) GO and KEGG enrichment analysis of DEGs in the hypothalamus. (B) GO and KEGG enrichment analysis of DEGs in the pituitary. In panels (A) and (B), the size of the circles represents the gene count enriched in each pathway, and the color gradient from blue to red represents statistical significance (-log_10_(*p*-value)). Representative core genes in each term are listed under the respective pathway names. (C) Multi-GSEA enrichment curves for representative KEGG pathways in the hypothalamus, including Neuroactive ligand-receptor interaction (ko04080), GnRH signaling pathway (ko04912), Calcium signaling pathway (ko04020), and MAPK signaling pathway (ko04010). (D) Individual GSEA mountain plots showing representative core pathways in the pituitary, including Cortisol synthesis and secretion (upregulated), Ribosome, Oxidative phosphorylation, and Apoptosis - multiple species (downregulated).

Downstream in the pituitary, DEGs reflected a clear physiological prioritization: maximizing reproductive hormone synthesis while ensuring cell survival. Conventional KEGG and GO analyses highlighted strong enrichment in gonadotropins, receptors, and cell proliferation machinery (*PRL, FSHB, CGA, TRHR, SOX2, FOXO6*; Fig. 3C; Table S9). Complementary GSEA revealed a profound metabolic and cell-survival adaptation (Fig. 3D): while apoptosis was significantly suppressed (NES = −1.57) to preserve secretory cell mass, fundamental translational machinery (Ribosome, NES = −2.40) and mitochondrial energy expenditure (Oxidative phosphorylation, NES = −1.84) were markedly downregulated (Table S10). This indicates a strategic shift in resource allocation, trade-offs in basal maintenance, and suppression of cell death to sustain high secretory output.

Together, the central hypothalamic-pituitary axis orchestrates peak laying performance through a hierarchy of events: the hypothalamus integrates environmental circadian cues and energy status to signal via neuropeptides (*NPY, VIP*), which in turn triggers pituitary receptor networks, neuropeptide/gonadotropic hormone synthesis (*CGA, PRL, FSHB, PRLH*), and downstream endocrine feedback signaling. Concurrently, the pituitary suppresses apoptotic pathways and restructures basic translational and energy consumption, ensuring prolonged survival and robust hormone synthesis to directly support follicle maturation.

#### Peripheral metabolic and ovarian adaptations: ovary-liver

To elucidate the peripheral tissue dynamics driven by central endocrine inputs, functional enrichment analysis was performed on DEGs in the ovary and liver. In the ovary, functional profiling demonstrated a clear developmental switch from early cell fate commitment to rapid tissue expansion and nutrient reception. Enrichment analyses underscored extensive extracellular matrix restructuring (*ADAMTS1, COL1A1/2*; ECM-receptor interaction NES = 2.29), active vascularization (*PECAM1, TEK*), and steroidogenesis (*STAR, INHBB, PRL*; Fig. 4A, B; Table S11). Activation of crucial survival and endocrine cascades (PI3K-Akt, Relaxin, and Estrogen signaling pathways) directly supported follicle survival during rapid yolk uptake, whereas early developmental pathways such as Notch signaling (NES = −1.83) were markedly suppressed (Table S12).

**Fig. 4:**
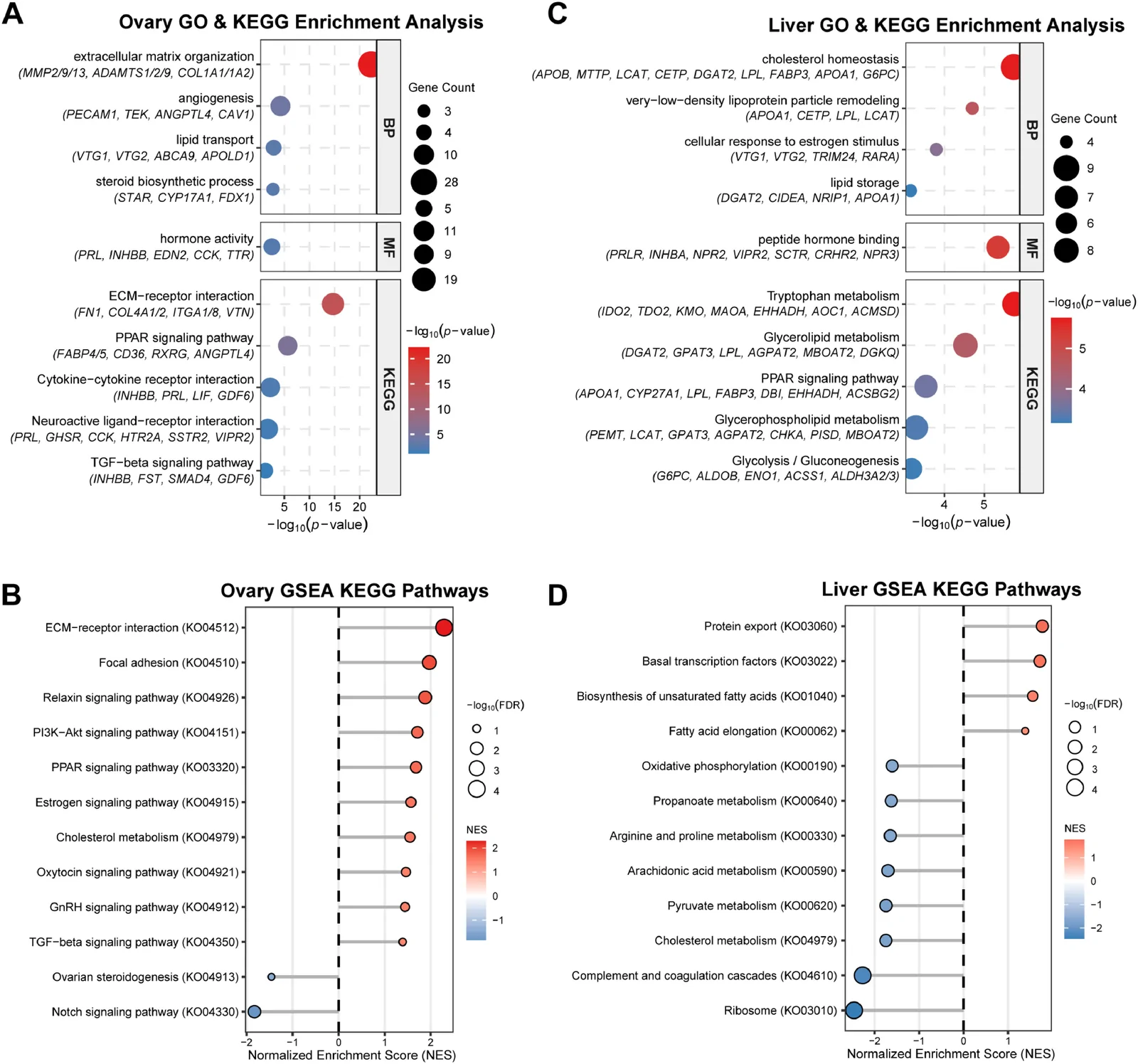
**Functional enrichment and gene set enrichment analysis (GSEA) of DEGs in the ovary and liver during the peak-laying period**. (A) GO terms (Biological Process, BP; Molecular Function, MF) and KEGG pathways enriched in the ovary. (B) GSEA lollipop plot showing 12 key enriched KEGG pathways in the ovary. (C) GO terms and KEGG pathways enriched in the liver. (D) GSEA lollipop plot showing 12 key enriched KEGG pathways in the liver. In panels A and C, the x-axis represents -log_10_(*P*-value), dot size corresponds to the number of enriched DEGs (Gene Count), and dot color indicates statistical significance (-log_10_(*P*-value)). Representative genes associated with each term or pathway are listed under the respective pathway name. In panels B and D, the x-axis represents the Normalized Enrichment Score (NES) derived from threshold-independent GSEA (with positive NES indicating enrichment in peak-laying hens [peakL] and negative NES indicating enrichment in pre-laying hens [preO/preL]). Dot size corresponds to -log_10_(FDR) and dot color denotes the NES value (red for positive enrichment, blue for negative enrichment).

In the liver, functional signatures unveiled a specialized metabolic reconfiguration dedicated to egg yolk precursor synthesis. Under central estrogenic stimulation (*VTG1/2, TRIM24*), the liver prioritized lipid handling and secretion (Fig. 4C, D; Table S13), upregulating fatty acid elongation, lipid storage (*APOB, DGAT2, LPL*), and protein export machinery (NES = 1.77) to process and dispatch VLDL and vitellogenin. To accommodate this heavy anabolic demand, the liver selectively suppressed basal translational machinery (Ribosome, NES = −2.46), immune surveillance (Complement cascades, NES = −2.27), and steady-state nutrient catabolism, effectively reallocating metabolic resources toward yolk precursor production (Table S14).

Together, these integrated analyses demonstrate a highly coordinated peripheral coupling: the liver functions as a specialized anabolic factory reallocating basal metabolic resources toward yolk precursor synthesis, while the ovary acts as an active metabolic sink, undergoing massive ECM remodeling, angiogenesis, and cell survival to accommodate rapid follicle growth.

### Construction of global WGCNA co-expression network and topological analysis of module relationships

To systematically identify key gene co-expression networks coordinating multi-tissue crosstalk and egg production performance, a global WGCNA was constructed using the transcriptome profiles of 24 RNA-seq samples (hypothalamus, pituitary, ovary, and liver tissues). We first analyzed the network topology to select an optimal soft-thresholding power (*β*). As shown in Fig. 5A, the scale-free fit index (*R*^2^) exceeded the standard evaluation threshold (*R*^2^ > 0.85) as *β* increased, while the mean connectivity steadily declined to a stable level, confirming that the constructed co-expression network strictly adhered to a scale-free topology.

**Fig. 5:**
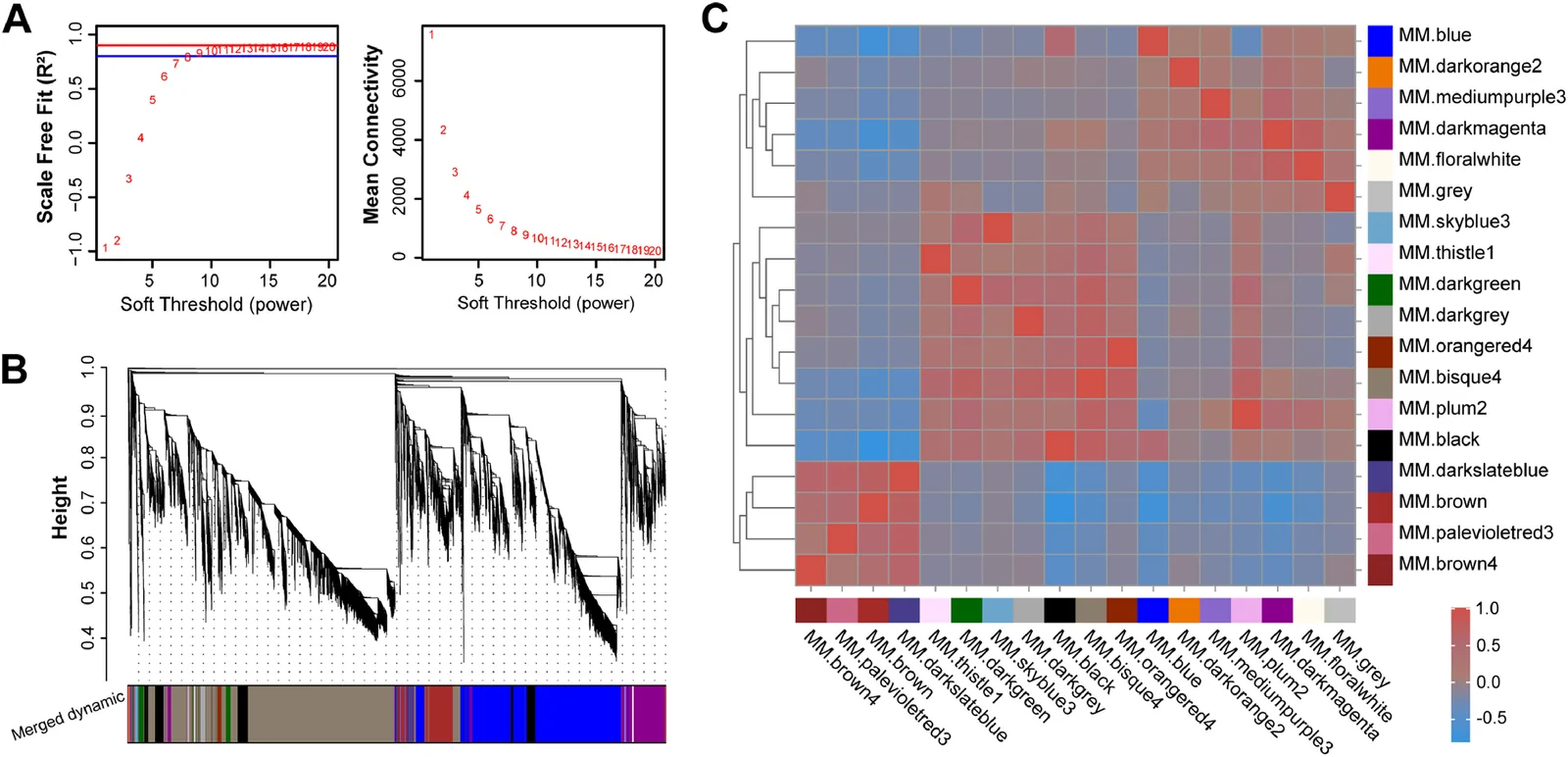
**Construction of the global WGCNA co-expression network and topological analysis of module relationships**. (A) Analysis of network topology for various soft-thresholding powers (*β*). The left panel shows the scale-free fit index (*R*^2^) as a function of soft-thresholding powers, with the red horizontal line indicating the fit evaluation threshold. The right panel shows the corresponding mean connectivity. (B) Gene dendrogram obtained by hierarchical clustering based on topological overlap matrix (TOM) dissimilarity (1-TOM). The color bands below indicate the final merged co-expression modules. (C) Heatmap of module eigengene (ME) correlations. The heatmap illustrates the global correlation matrix among the identified modules. Color scale ranges from blue (negative correlation) to red (positive correlation), depicting the synergistic and antagonistic relationships among functional modules.

Using the selected power parameter (*β* = 8), hierarchical clustering was performed based on TOM dissimilarity, and co-expression modules were defined using the dynamic tree cut algorithm, yielding a total of 18 distinct modules (Fig. 5B). To further elucidate the global topological architecture and inter-module crosstalk, we calculated the correlation matrix of MEs. The ME correlation heatmap revealed clear hierarchical clustering patterns and inter-module regulatory relationships, with specific module pairs exhibiting prominent synergistic (positive) or antagonistic (negative) co-expression dynamics (Fig. 5C). These results indicate strong functional specialization and tight co-regulation among the identified modules.

Additionally, the tissue-specific expression patterns of these 18 modules across all 24 individual samples are illustrated in Supplementary Fig. S1. Global module-trait association analysis (Fig. S2A) combined with average GS profiling (Fig. S3) identified highly specific signature modules for each tissue (Table S15−S19). In contrast, stage-related correlation (pre-laying vs. Peak-laying) was primarily concentrated in smaller, highly specialized sub-modules (Fig. S2A and Fig. S3E; Table S14), suggesting that the peak-laying transition is dictated by inter-tissue functional synergy rather than broad single-module dominant shifts.

#### Identification of tissue-specific hub genes and functional regulatory networks

To systematically dissect the molecular architecture driving tissue-specific physiological functions, we prioritized the top signature module for each tissue based on module-trait associations. Strong positive correlations between MM and GS within these key tissue modules (*r* ≥ 0.76, P = 1 × 10^−200^; Fig. S2B) demonstrated that intra-modular hub genes are highly representative of tissue-specific physiological features. The integration of hub gene regulatory networks with downstream KEGG pathway enrichment analyses of these corresponding signature modules revealed profound functional specialization across organs (Table S20–S23).

In the hypothalamus, functional enrichment of the hypothalamic signature (Blue module) highlighted significant pathways including Adrenergic signaling in cardiomyocytes, Calcium signaling pathway, and Cell adhesion molecules (CAMs), establishing the molecular foundation for central rhythm output and neurotransmitter release. These central network processes were topologically governed by core hub genes including *ADGRL3, NRXN1*, and *SYT11* (Fig. 6A). Concurrently, the pituitary signature (Darkmagenta) module underscored pathways such as Endocytosis and Glycosphingolipid biosynthesis, which are essential for hormone synthesis and secretory vesicle dynamics, driven by central network nodes including *EYA1, EYA2, DACH1*, and *PAX6* (Fig. 6B).

**Fig. 6:**
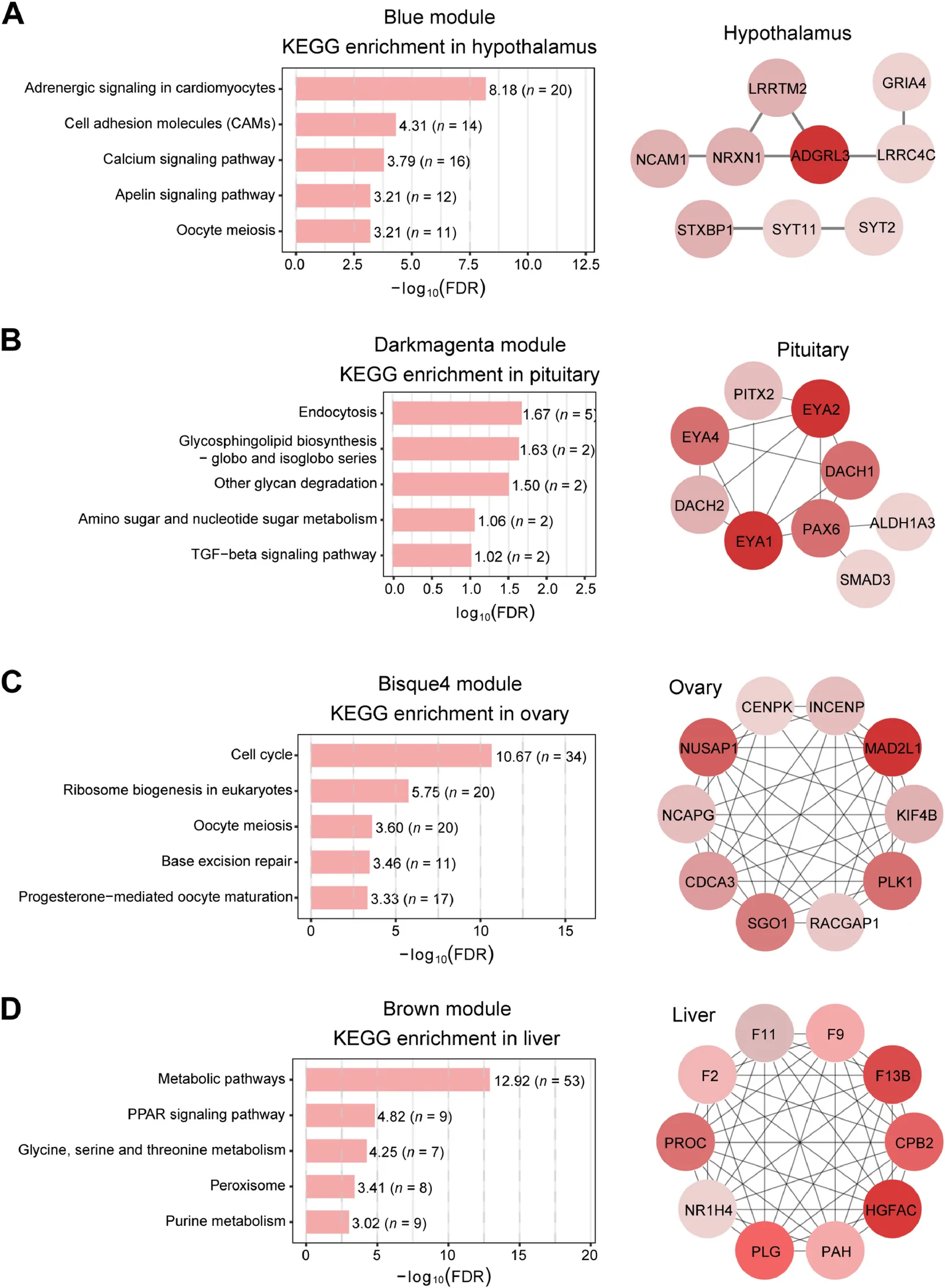
**Functional pathway enrichment and core protein-protein interaction (PPI) networks in tissue-specific modules**. (A) Hypothalamus, (B) Pituitary, (C) Ovary, and (D) Liver. For each organ, functional pathway enrichment of the corresponding co-expression module is presented on the left, with bar plots displaying the X-axis representing the -log_10_(FDR) value of enrichment and numbers in parentheses indicating the count of input genes (*n*) enriched in each pathway. The core PPI network is presented on the right, where node colors denote the maximal clique centrality (MCC) ranking (with dark to medium red indicating highest-scoring core hubs, and lighter red to pale pink representing secondary nodes).

Within the reproductive and metabolic systems, functional enrichment in the ovarian signature (Bisque4) module pointed to key reproductive and cell-cycle pathways including Cell cycle, Ribosome biogenesis in eukaryotes, Oocyte meiosis, and Progesterone-mediated oocyte maturation, coordinately regulated by core hub regulators such as *PLK1, MAD2L1, INCENP*, and *EYA2* (Fig. 6C). Finally, the hepatic signature (Brown) module demonstrated prominent involvement in Metabolic pathways and PPAR signaling pathway, reaffirming the liver's primary regulatory role in systemic lipid and energy homeostasis, which were anchored by core driver genes including *F2, PROC, PLG, HGFAC, PAH*, and *NR1H4* (FXR) (Fig. 6D).

By demonstrating that these key hub genes are directly integrated within their respective enriched KEGG and GO pathway terms, our findings confirm that these central regulators not only possess high topological connectivity within the networks, but also actively drive the core biochemical pathways governing tissue-specific physiological specialization. To experimentally validate these networks, representative hub genes were prioritized based on a combined criterion of network topological centrality and transcriptional dynamics. Accordingly, key candidate hubs, including *NRXN1* for the hypothalamus, *EYA1* for the pituitary, *PLK1* for the ovary, and *PAH* for the liver (Table S24), were selected for downstream qPCR analysis to robustly bridge network architecture with experimental confirmation.

#### Integration of inter-module crosstalk and multi-stage ligand-receptor communication networks

To link global co-expression modules with intercellular communication, module eigengene correlations were integrated with a multi-stage ligand-receptor network (Fig. 7). Module correlation analysis among the hypothalamus, pituitary (Pituitary_darkmagenta), ovary, and liver signature modules revealed that central neuroendocrine and gonadal modules exhibit positive or neutral correlations (e.g., *r* = 0.26 between hypothalamus and pituitary), reflecting synchronized activation (Fig. 7A). Conversely, the hepatic module showed robust negative correlations with the hypothalamus (*r* = −0.68, *P* < 0.001), pituitary (*r* = −0.63, *P* < 0.01), and ovary (*r* = −0.48, *P* < 0.05), capturing the systemic metabolic trade-off required to redirect energy toward hepatic yolk precursor synthesis.

**Fig. 7:**
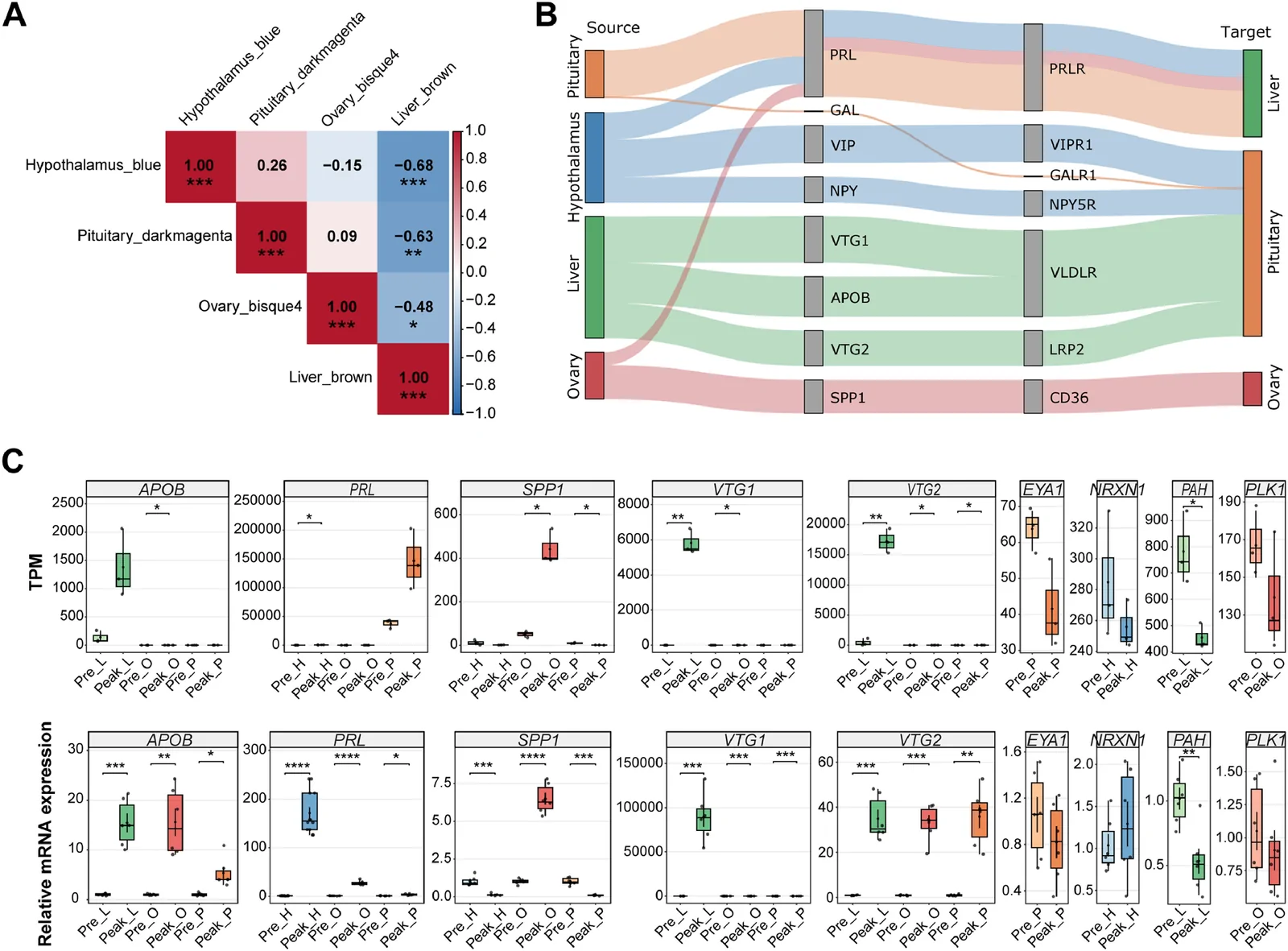
**Integration of inter-module crosstalk, multi-stage ligand-receptor communication networks, and quantitative gene expression validation**. (A) Pearson correlation heatmap of module eigengenes among hypothalamus, pituitary, ovary, and liver signature modules. Positive and negative correlations are indicated by red and blue gradients, respectively. Asterisks denote statistical significance (*P < 0.05, **P < 0.01, ***P < 0.001). (B) Multi-stage Sankey diagram mapping expression-inferred intercellular communication flows from source tissues through key ligands and receptors to target tissues (flow widths proportional to peak-laying co-expression weights). (C) Expression levels (TPM, top panel) and relative mRNA expression verified by quantitative real-time PCR (qPCR, bottom panel) for 5 key candidate genes (*APOB, PRL, SPP1, VTG1*, and *VTG2*) across hypothalamus (H), liver (L), ovary (O), and pituitary (P) tissues, alongside four representative tissue-specific hub genes (*NRXN1* for hypothalamus, *EYA1* for pituitary, *PLK1* for ovary, and *PAH* for liver) at pre-laying (Pre) and peak-laying (Peak) stages. Asterisks indicate significant differences between Pre and Peak stages within each tissue (*P < 0.05, **P < 0.01, ***P < 0.001, Student's t-test). Nonsignificant comparisons are unlabeled.

A multi-stage Sankey network mapped precise intercellular communication flows across source tissues, ligands, receptors, and target tissues (Fig. 7B). This network highlights major cross-tissue axes, including systemic *PRL* from the hypothalamus, pituitary, and ovary targeting hepatic *PRLR*, alongside hepatic-derived *VTG1, VTG2*, and *APOB* feeding back to pituitary receptors (*VLDLR* and *LRP2*). Additionally, local circuits (*NPY*−*NPY5R, VIP*−*VIPR1, GAL*−*GALR1*, and *SPP1*−*CD36*) fine-tune reproductive activation. Together, these results demonstrate that the onset of lay is governed by a synchronized hierarchy linking central neuroendocrine commands with peripheral anabolic factories via precise ligand−receptor signaling.

#### Validation of key candidate gene expression profiles

To validate the robustness and reliability of the transcriptomic quantification and substantiate the key functional hubs identified across both co-expression networks and ligand–receptor axes, qPCR was performed on core functional candidate genes (*APOB, PRL, SPP1, VTG1*, and *VTG2*) alongside representative tissue-specific hub genes prioritized in Table S23 (*NRXN1* for hypothalamus, *EYA1* for pituitary, *PLK1* for ovary, and *PAH* for liver) between pre-laying and peak-laying stages (Fig. 7C).

The relative mRNA expression patterns measured by qPCR exhibited high concordance with the RNA-seq TPM profiles (Fig. 7C). Specifically, core candidate genes demonstrated dramatic stage-dependent changes: *PRL* showed marked elevation in the pituitary at peak-lay, hepatic yolk precursor genes (*APOB, VTG1*, and *VTG2*) displayed robust multi-fold upregulation, and ovarian *SPP1* was significantly upregulated (*P* < 0.05). Among the representative tissue-specific hub genes prioritized in Table S23, hepatic *PAH* exhibited a statistically significant decrease at peak-lay (*P* < 0.01), corroborating its dynamic metabolic driver role, whereas *NRXN1, EYA1*, and *PLK1* displayed expression trajectories consistent with RNA-seq trends (*P* > 0.05; Fig. 7C). Overall, this high level of consistency reinforces the accuracy of our global transcriptomic profiling and solidifies the biological validity of the primary regulatory drivers governing the onset of lay.

## Discussion

The onset of lay is a systemic physiological transition that couples neuroendocrine command with peripheral metabolic capacity. Indigenous breeds such as Wuhua Yellow chickens retain largely unselected genetic backgrounds and comparatively modest annual egg output, making them informative models for deciphering the native architecture of reproductive–metabolic coordination (Wang et al., 2024; Huang et al., 2025). Here we integrated multi-tissue transcriptomes from the hypothalamus, pituitary, ovary and liver at pre-laying and peak-laying stages, combining with WGCNA, ligand–receptor inference and rigorous qPCR validation. Our integrated framework proposes that the pre- to peak-lay transition is governed by hierarchical inter-tissue crosstalk along an extended hypothalamic–pituitary–ovary–liver (HPO–liver) axis rather than by isolated organ programs (Fig. 8).

**Fig. 8:**
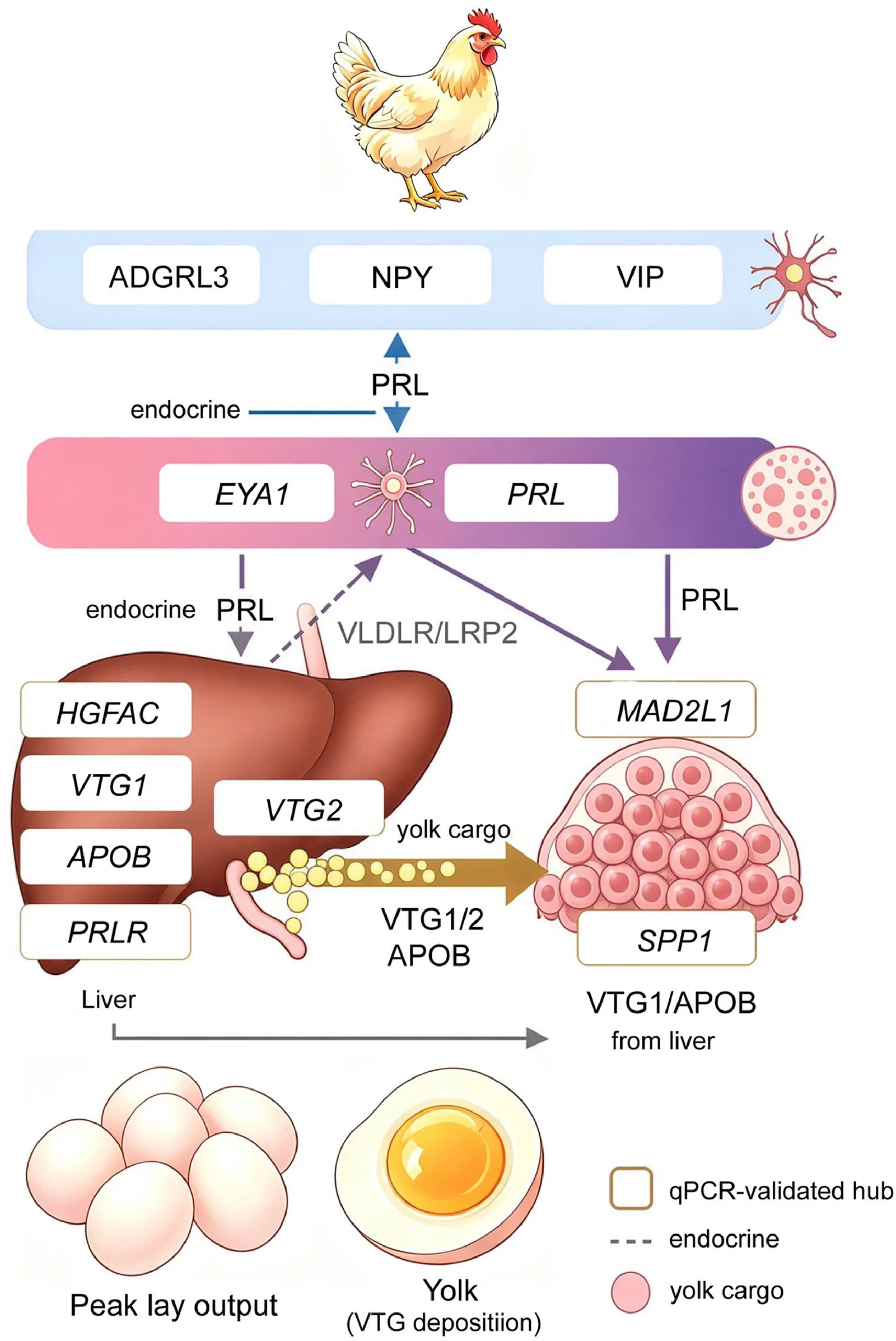
**Integrative summary of the HPO–liver axis coordinating the onset of lay in Wuhua Yellow chickens**. Central hypothalamic signals (represented by *ADGRL3, NPY*, and *VIP*) and pituitary endocrine output (*PRL*, with hub *EYA1*) drive hepatic yolk-precursor synthesis via PRLR. The liver produces VTG1, VTG2, and APOB, which are transported as yolk cargo to the ovary (hubs *MAD2L1* and *SPP1*). Dashed lines indicate expression-inferred endocrine routes, including potential feedback via *VLDLR*/*LRP2*. Boxes with borders denote qPCR-validated hubs. The diagram summarizes the systemic transcriptional coordination supporting peak lay.

### A systemic ligand–receptor axis links central endocrine output to hepatic yolk precursor production

A central observation of this study is the coordinated upregulation of *PRL* across multiple nodes of the HPO-liver axis, concurrent with the dramatic induction of its receptor in the liver. Hypothalamic *PRL* increased by log₂FC = 7.77, pituitary *PRL* by log_2_FC = 1.95 and ovarian *PRL* by log_2_FC = 4.83, while hepatic *PRLR* rose by log_2_FC = 4.23. Concurrently, the liver exhibited extreme induction of the principal yolk precursors: *VTG1* (log_2_FC = 18.28), *VTG2* (log_2_FC = 6.60) and *APOB* (log_2_FC = 3.31). These magnitudes place yolk-precursor genes among the most dynamically regulated transcripts in the entire data set and are consistent with the classical view of the laying-hen liver as a specialized secretory organ (Gloux et al., 2019; Ren et al., 2021) and aligning with recent multi-tissue surveys that repeatedly identify *PRL*, vitellogenins and apolipoproteins as stage- or yield-responsive genes (Shi et al., 2024; Wang et al., 2024; Bai et al., 2025; Han et al., 2025). Crucially, the marked hepatic *PRLR* induction further supports a model in which prolactin signaling, in addition to canonical estrogen action, actively contributes to the anabolic program at the onset of lay (Gloux et al., 2019).

The ovary displayed a complementary “sink” signature. *VTG2* (log_₂_FC = 5.93) and *APOB* (log_2_FC = 4.89) were strongly upregulated in ovarian tissue, accompanied by extracellular-matrix remodeling genes such as *MMP13* (log_2_FC = 5.20) and collagens, consistent with structural preparation for rapid yolk uptake and hierarchical follicle growth (Leng et al., 2024; Wang et al., 2024). In parallel, downregulation of pituitary *VLDLR* (log_2_FC = −1.64) and *LRP2* (log_2_FC = −5.91), receptors involved in lipoprotein and vitellogenin handling, is compatible with a possible expression-level feedback route from peripheral metabolic status toward the central endocrine axis. Together, these transcript patterns outline a putative HPO–liver communication scheme in which central and local *PRL* induction coincides with hepatic precursor synthesis, precursors are available for ovarian uptake, and reduced pituitary lipoprotein-receptor expression may reflect sensing of peripheral lipid/yolk-precursor load. Direct hormone measurements and functional tests will be required to confirm this inferred circuitry.

Local neuromodulatory circuits further refine the extended axis. Hypothalamic and pituitary *NPY* were elevated (log_2_FC = 1.09 and 1.86, respectively), while pituitary *NPY5R* declined, consistent with the established role of neuropeptide Y in linking energy balance to reproductive drive in birds (Greene et al., 2022; Wang et al., 2024). Expression-inferred changes in *VIP*–*VIPR* and *GAL*–*GALR1* pairs, together with ovarian *SPP1*–*CD36* co-induction (supported by stage-dependent upregulation of both partners), point to additional fine-tuning of steroidogenic, matrix and lipid-handling processes. Reflecting this systemic integration at the macro-nework level, module-eigengene correlations supply an independent topological corroboration: the hepatic Brown module was negatively correlated with the hypothalamic Blue (*r* = −0.68, *P* < 0.001), pituitary Darkmagenta (*r* = −0.63, *P* < 0.01) and ovarian Bisque4 (*r* = −0.48, *P* < 0.05) modules. This antagonistic relationship neatly captures a systemic metabolic trade-off that reallocates transcriptional and energetic resources toward massive yolk synthesis at the expense of other transcriptional programs (Shi et al., 2024; Wang et al., 2024; Bai et al., 2025).

Highlighting the global nature of this coordination, five genes, including *VTG2*, were differentially expressed in all four tissues, underscoring a tightly integrated systemic response. The hypothalamus and pituitary shared 484 DEGs, reflecting coordinated neuroendocrine remodeling, while a smaller set of 24 genes was jointly regulated across the hypothalamus–pituitary–ovary triad. Although ligand–receptor relationships inferred from transcript abundance remain correlative, the strong concordance between RNA-seq and qPCR for key candidates (*PRL, VTG1, VTG2, APOB,* and *SPP1*) across tissues strengthens confidence in these core signaling axes. Orthogonal assays of representative tissue hubs further illustrate effect-size heterogeneity across the HPO–liver network: hepatic *PAH* was robustly confirmed as down-regulated at peak-lay, whereas smaller, context-dependent hubs (*NRXN1, EYA1*, and *PLK1*) largely tracked RNA-seq trends without reaching significance in the expanded qPCR set. Thus the multi-tissue expression dynamics themselves delineate a coordinated neuroendocrine–metabolic network governing the onset of lay in this indigenous population, in line with recent multi-tissue surveys of the avian HPO–liver axis (Wang et al., 2024; Zhu et al., 2025).

### Tissue-specific co-expression hubs encode functional division of labor

Global WGCNA of 24 WYC samples yielded 18 modules under a soft-thresholding power of *β* = 8 that satisfied scale-free topology (*R*^2^ > 0.85). Signature modules for each tissue exhibited strong positive correlations between MM and GS (*r* ≥ 0.76, *P* = 1 × 10^−200^), indicating that intra-modular hubs faithfully represent tissue-specific physiological features (Langfelder and Horvath, 2008; Wang et al., 2024).

In the hypothalamus the Blue-module hubs *ADGRL3, NRXN1* and *SYT11* reside within pathways related to cell adhesion, calcium signaling and synaptic function, consistent with a role in sensing photoperiodic, energetic and neuroendocrine inputs and in driving downstream outputs (Leng et al., 2024; Wang et al., 2024). Notably, consistent with its central topological role, *NRXN1* has also been recovered among egg-production candidates in a same-breed GWAS (Huang et al., 2025). Pituitary Darkmagenta hubs centered on *EYA1* (together with *EYA2, DACH1* and *PAX6*) coincide with enrichment for endocytosis and glycosphingolipid biosynthesis, processes required for sustained hormone packaging and release. The pituitary itself showed the largest DEG count (3,419 genes), reflecting a major functional specialization toward reproductive hormone synthesis; concurrent GSEA indicated suppression of apoptosis (NES = −1.57) and of basal translational and oxidative-phosphorylation machinery, suggesting a strategic prioritization of secretory output over cellular maintenance (Chen et al., 2024; Li et al., 2024; Shi et al., 2024).

Ovarian Bisque4 hubs such as *MAD2L1, PLK1* and *INCENP* anchor cell-cycle, oocyte-meiosis and progesterone-mediated maturation pathways that enable rapid follicular expansion (Leng et al., 2024; Yuan et al., 2025). Parallel enrichment of ECM-receptor interaction, angiogenesis and PI3K–Akt / estrogen signaling supports follicle survival during intense yolk deposition, while early developmental programs such as Notch signaling were suppressed. Hepatic Brown-module hubs including *HGFAC, F2, PROC, PLG* and *NR1H4* (FXR) align with metabolic pathways and PPAR signaling, reaffirming the liver’s role as the primary site of systemic lipid and energy homeostasis dedicated to yolk-precursor output (Gloux et al., 2019; Ren et al., 2021; Shi et al., 2024). Genes linked to hepatic lipid handling and energy partitioning in our network, such as *MTTP* and *PPARA*, likewise appear among same-breed GWAS candidates for egg-production traits (Huang et al., 2025), providing ancillary genetic support for the metabolic arm of the axis. GSEA of hepatic DEGs further revealed selective down-regulation of ribosomal and complement cascades, consistent with the reallocation of metabolic resources away from basal maintenance and immune surveillance toward anabolic secretion. Beyond the lipid-secretory program centered on *VTG*/*APOB*, hepatic amino-acid metabolism is also expected to be remodeled when anabolic capacity is redirected to yolk precursor production (Gloux et al., 2019; Ren et al., 2021). In this context, the significant peak-lay decline of *PAH* (phenylalanine hydroxylase) is compatible with attenuated basal catabolism of aromatic amino acids as transcriptional and metabolic resources shift toward *VTG*/*APOB*-centered output (Ren et al., 2021; Shi et al., 2024). Such a pattern supports a broader view of the liver module as a multi-pathway anabolic factory, not limited to lipoprotein genes alone (Gloux et al., 2019; Wang et al., 2024).

Collectively, these tissue-specific hubs and their associated pathways delineate an integrated division of labor across the HPO–liver axis, in which central sensing and endocrine output are tightly coupled to peripheral anabolic production and ovarian structural–metabolic reception, thereby coordinating the systemic transition into peak lay. The negative module correlations between the liver and the central/gonadal modules indicate that this division is not merely additive but is organized as a systemic trade-off at the onset of peak lay.

### Limitations

Several constraints should be acknowledged. RNA-seq used *n* = 3 per tissue–stage, which is adequate for network discovery of large effects but limited for subtle signals; qPCR on *n* = 6 partially mitigates this for prioritized genes. Ovarian sampling differed by stage (whole ovary at pre-lay versus small yellow follicles at peak-lay), which may contribute to ovary-specific DEGs independent of systemic signals. Ligand–receptor links were inferred from expression after mapping human/mouse CellChatDB pairs to chicken orthologs and were not validated by circulating PRL, estrogen, or VTG protein assays or receptor perturbation. Pituitary *VTG1*/*VTG2* induction, though directionally consistent with systemic change, remains far below hepatic levels and needs protein- and cell-type-level confirmation (Leng et al., 2024). Only two stages were compared, so the temporal order of inter-tissue signaling is unresolved. A further limitation is that bone tissue was not sampled; thus, potential distal effects of hypothalamic or pituitary secreted factors on medullary bone remodeling could not be assessed. Finally, transcriptomic hubs and same-breed genetic signals are complementary; the present study identifies expression-level candidates rather than sequence variants, and eQTL or multi-omics integration will be required for causal inference (Huang et al., 2025; Zhu et al., 2025).

### Implications for breeding and management

The HPO–liver ligand–receptor axes (*PRL*–*PRLR, VTG1*/*VTG2*/*APOB*) and qPCR-confirmed nodes (*PRL, VTG1, VTG*2, *APOB, SPP1*, and hepatic *PAH*), together with hubs that showed concordant RNA-seq/qPCR trends (*NRXN1, EYA1*, and *PLK1*), nominate prioritized expression-level candidates for further genetic and functional validation, with potential relevance to earlier or more sustained peak lay in Wuhua Yellow chickens while preserving genetic diversity and meat quality (Wang et al., 2024; Huang et al., 2025). Combining these expression-level candidates with existing GWAS loci may, after association testing in larger populations, help refine selection indices for dual-purpose indigenous stocks. From a management perspective, the hepatic anabolic signature and central energy-sensing transcripts (*NPY*) provide a molecular rationale for optimizing pre-peak nutrition and photoperiod to support the metabolic transition into peak lay (Greene et al., 2022). The multi-tissue framework is transferable to other low-producing indigenous breeds in which reproduction–metabolism balance, rather than maximal output alone, is the breeding goal. Association studies, circulating hormone assays, and targeted functional tests will be required before these network-derived candidates can inform practical breeding decisions.

In summary, the onset of lay in Wuhua Yellow chickens emerges as a synchronized neuroendocrine–hepatic–ovarian dialogue. By inferring both the intercellular communication routes and the intra-tissue hub architecture that implement this dialogue, the present study advances a systems-level understanding of reproductive activation in an indigenous chicken and highlights prioritized expression-level candidates for further validation toward sustainable improvement of egg production.

## Funding

This work was supported by Peak Talent Program of Jiaying University (2022RC45), the Scientific Research Innovation Team Project of Jiaying University (2021JYUTDS04), Jiaying University 2026 Undergraduate Innovation Training Program Project (to Yi-xuan Wang), and Jiaying University 2026 Smart Course Construction Project: General Biology (to Yong-jie Xu).

## Availability of data and materials

The sequencing data have been deposited in the NCBI Sequence Read Archive under the BioProject accession number PRJNA1504820.

## Author contributions

Yong-jie Xu conceived and designed the study, developed the analytical framework, performed the multi-tissue transcriptomic and network analyses, interpreted the results, prepared the Figs. and tables, and drafted the original manuscript. Yi-xuan Wang contributed to data validation, Fig. preparation and visualization, and critically reviewed the manuscript for important intellectual content. Zhuo-xian Weng contributed to data processing, bioinformatic analysis, network construction, and visualization, and critically reviewed the manuscript. Yu-nan Zou contributed to experimental validation and data checking, and critically reviewed the manuscript. Wei-na Li and Jie-bo Chen contributed to animal management, tissue sampling, and sample preparation, and critically reviewed the manuscript. Bing-wang Du contributed to the interpretation of results and the discussion of biological implications, and critically revised the manuscript. Xun-he Huang conceived and designed the study, supervised the overall research, secured funding, interpreted the data, and critically revised the manuscript. All authors critically reviewed the manuscript for important intellectual content, approved the final version to be published, and agree to be accountable for all aspects of the work, ensuring that any questions related to the accuracy or integrity of any part of the research are appropriately investigated and resolved.

## Disclosures

The authors declare that they have no known competing financial interests or personal relationships that could have appeared to influence the work reported in this paper.

## Footnotes

Supplementary material associated with this article can be found, in the online version, at doi:10.1016/j.psj.2026.108073.
