# Supplementary material for: Integrated multi-tissue transcriptomic and network analyses reveal systemic crosstalk governing the onset of lay in Wuhua Yellow chickens
Source: Poult Sci. 2026 Oct 31;105(12):108073. doi: 10.1016/j.psj.2026.108073 (PMC13760473; doi:10.1016/j.psj.2026.108073)
Supplement: Supplementary file 1 [file mmc1.docx]

**Supplementary figures**

**Integrated multi-tissue transcriptomic and network analyses reveal systemic crosstalk governing the onset of lay in Wuhua Yellow chickens**

Yong-jie Xu, Yi-xuan Wang, Zhuo-xian Weng, Yu-nan Zou, Wei-na Li, Jie-bo Chen, Bing-wang Du, and Xun-he Huang^*^

*Guangdong Provincial Key Laboratory of Conservation and Precision Utilization of Characteristic Agricultural Resources in Mountainous Areas, Guangdong Innovation Centre for Science and Technology of Wuhua Yellow Chicken, School of Life Sciences, Jiaying University, Meizhou 514015, China*

* Corresponding author

E-mail address: hxh826@jyu.edu.cn (X.H. Huang)

**Figure S1. Module eigengene (ME) expression patterns across 24 samples from four tissues at pre-laying and peak-laying stages.** Heatmap illustrating the ME expression levels of 18 co-expression modules across individual samples. Sample labels indicate developmental stage (Pre or Peak), tissue type (H: Hypothalamus, P: Pituitary, O: Ovary, L: Liver), and biological replicate numbers (1–3). Row hierarchical clustering on the left groups modules with similar expression trends across samples. The color bar indicates scaled ME expression values, where red corresponds to high expression and blue corresponds to low expression.

**Figure S2. Module-trait relationships and correlation between module membership (MM) and gene significance (GS) across tissues.** (A) Heatmap of module-trait relationships showing the correlation coefficients between module eigengenes (MEs) and traits/tissues (Stage_Peak, Tissue_Hypothalamus, Tissue_Liver, Tissue_Ovary, and Tissue_Pituitary). Color gradient represents Pearson correlation coefficients ranging from -0.5 (blue) to 0.5 (red). Statistical significance is indicated by asterisks: **P* < 0.05, ***P* < 0.01, and ****P* < 0.001. (B) Scatter plots of MM versus GS for representative modules highly associated with each of the four tissues (Hypothalamus, Pituitary, Ovary, and Liver). Each dot represents a gene within the respective tissue-specific module. Pearson correlation coefficients (cor) and corresponding *P*-_values_ (*p*) are indicated at the top of each panel, demonstrating strong intra-modular co-regulation for key tissue-specific networks.

**Figure S3. Association of WGCNA co-expression modules with specific tissues and the egg production stage.** Bar plots display the average gene significance (GS) across the 18 identified modules for each trait: (A) Hypothalamus, (B) Pituitary, (C) Ovary, (D) Liver, and (E) Egg production stage (Stage_Peak). GS is defined as the absolute value of the correlation coefficient between gene expression and the given trait. Bars are colored according to the WGCNA module colors. Error bars represent standard errors. Key tissue-specific or stage-associated modules can be identified by the highest bar values in each panel.
